# Toll-Like Receptor 4 on both Myeloid Cells and Dendritic Cells Is Required for Systemic Inflammation and Organ Damage after Hemorrhagic Shock with Tissue Trauma in Mice

**DOI:** 10.3389/fimmu.2017.01672

**Published:** 2017-11-28

**Authors:** Kent Zettel, Sebastian Korff, Ruben Zamora, Adrian E. Morelli, Sophie Darwiche, Patricia A. Loughran, Greg Elson, Limin Shang, Susana Salgado-Pires, Melanie J. Scott, Yoram Vodovotz, Timothy R. Billiar

**Affiliations:** ^1^Department of Surgery, University of Pittsburgh, Pittsburgh, PA, United States; ^2^Department of Trauma Surgery, University of Heidelberg, Heidelberg, Germany; ^3^Novimmune SA, Geneva, Switzerland; ^4^Glenmark Pharmaceuticals SA, La-Chaux-de-Fonds, Switzerland

**Keywords:** trauma, toll-like receptor 4, dendritic cells, myeloid cells, hemorrhagic shock

## Abstract

Trauma combined with hemorrhagic shock (HS/T) leads to systemic inflammation, which results in organ injury. Toll-like Receptor 4 (TLR4)-signaling activation contributes to the initiation of inflammatory pathways following HS/T but its cell-specific roles in this setting are not known. We assessed the importance of TLR4 on leukocytes of myeloid lineage and dendritic cells (DCs) to the early systemic inflammatory response following HS/T. Mice were subjected to HS/T and 20 inflammatory mediators were measured in plasma followed by Dynamic Bayesian Network (DBN) Analysis. Organ damage was assessed by histology and plasma ALT levels. The role of TLR4 was determined using TLR4^−/−^, MyD88^−/−^, and Trif^−/−^ C57BL/6 (B6) mice, and by *in vivo* administration of a TLR4-specific neutralizing monoclonal antibody (mAb). The contribution of TLR4 expressed by myeloid leukocytes and DC was determined by generating cell-specific TLR4^−/−^ B6 mice, including Lyz-Cre × TLR4^loxP/loxP^, and CD11c-Cre × TLR4^loxP/loxP^ B6 mice. Adoptive transfer of bone marrow-derived TLR4^+/+^ or TLR4^−/−^ DC into TLR4^−/−^ mice confirmed the contribution of TLR4 on DC to the systemic inflammatory response after HS/T. Using both global knockout mice and the TLR4-blocking mAb 1A6 we established a central role for TLR4 in driving systemic inflammation. Using cell-selective TLR4^−/−^ B6 mice, we found that TLR4 expression on both myeloid cells and CD11c^high^ DC is required for increases in systemic cytokine levels and organ damage after HS/T. We confirmed the capacity of TLR4 on CD11c^high^ DC to promote inflammation and liver damage using adoptive transfer of TLR4^+/+^ conventional (CD11c^high^) DC into TLR4^−/−^ mice. DBN inference identified CXC chemokines as proximal drivers of dynamic changes in the circulating levels of cytokines/chemokines after HS/T. TLR4 on DC was found to contribute selectively to the elevations in these proximal drivers. TLR4 on both myeloid cells and conventional DC is required for the initial systemic inflammation and organ damage in a mouse model of HS/T. This includes a role for TLR4 on DC in promoting increases in the early inflammatory networks identified in HS/T. These data establish DC along with macrophages as essential to the recognition of tissue damage and stress following tissue trauma with HS.

## Introduction

Severe trauma combining systemic hypoperfusion with extensive tissue injury induces the rapid onset of a systemic inflammatory response and end-organ injury (1). Aspects of this early response can be replicated in murine models of hemorrhagic shock (HS) plus tissue trauma (HS/T) (2). For example, experimental models combining HS plus tissue trauma in mice lead to increases in plasma chemokines and cytokines within hours; as well as lung and liver damage that results from this mediator “storm” (2). The systemic inflammatory response induced by HS/T is thought to result from a surge in the release of damage-associated molecular pattern molecules (DAMPs) from stressed and damaged tissues (3). These DAMPs are sensed by receptors of the innate immune system that trigger immune system activation and production of inflammatory mediators (3). Strong experimental evidence now exists for the importance of toll-like receptors (TLR) such as toll-like receptor 4 (TLR4), TLR2, TLR9, and TLR3 (4–8) as DAMP sensors in the initial immune response to blunt tissue trauma. These TLR are expressed on a wide range of cell types and we have provided evidence that expression of TLR4, TLR2, and TLR9 on both bone marrow (BM)-derived and non-BM-derived cells contribute to the systemic inflammatory response and liver damage in HS and tissue trauma models (9, 10).

Among the TLR involved in sterile inflammation, TLR4 appears to be the most promiscuous in its recognition of DAMPs (3). TLR4 recognizes a large repertoire of proteins, lipoproteins, and lipids from endogenous sources (5, 11–15). This leads to the activation of inflammatory signaling pathways that account for the wide range of sterile inflammatory conditions in which TLR4 is involved (16–22). Indeed, we have demonstrated that TLR4 on platelets contributes to microthrombi formation in a model of HS (23); furthermore, TLR4 on intestinal epithelial cells plays a role in intestinal inflammation that impacts lung injury following HS/T (24). These studies confirm that TLR4 expression on both BM-derived and non-BM-derived cells participate in pathogenesis of organ injury in these murine models of trauma and hemorrhage (9). However, the contribution of TLR4 on cells thought to be sentinels of the innate immune system for the “danger,” such as dendritic cells (DC) and macrophages, is not known.

Conventional DCs are a specialized subset of antigen-presenting cells that are known mostly for their role in regulating adaptive immunity and tolerance through the presentation of foreign or self-antigens (25, 26). However, evidence has begun to accumulate to suggest that DCs contribute to the immunoinflammatory response to acute systemic injury. More recently, a new subset of DC generated from blood monocytes and termed “inflammatory DCs” has been shown to participate in inflammatory/immune disorders. For example, DC numbers in peripheral blood fluctuate early after injury in humans, while monocyte-derived DCs show signs of early activation including notable changes in gene expression (27, 28). Studies in mouse models of HS/T have centered on the fate and function of splenocyte-derived DC, and have shown a loss of MHC class II expression and antigen presentation capacity early after injury in mice (26). Thus, DCs appear to be activated early after injury in both humans and mice. However, DCs express DAMP-sensing TLRs, including TLR4, and the role of these receptors on DC in regulating the initial inflammatory response following traumatic injury is not known.

Here, we examine the role of DC in TLR4-driven responses leading to systemic inflammation and end-organ damage in a model of severe HS/T in mice. We show that TLR4 deletion specifically from conventional (CD11c^high^) DC leads to a near complete inhibition of systemic increases in cytokines and chemokines as well as end-organ injury following HS/T. The TLR4-dependent responses from CD11c^high^ DC include the production of a group of CXC chemokines that were determined to be among the most proximal drivers of the early inflammatory response to systemic injury. Thus, populations of conventional DC are engaged early following injury through TLR4 to coordinate with other cell types, such as myeloid cells, platelets, and epithelial cells, the host inflammatory response to injury.

## Materials and Methods

### Reagents

All reagents were from Sigma-Aldrich Co. (St. Louis, MO, USA) unless otherwise indicated.

### Animals

This study was carried out in accordance with the recommendations of the National Institutes of Health Guidelines for the Use of Laboratory Animals. The protocol was approved by the Animal Care and Use Committee of the University of Pittsburgh. Male wild-type (WT) C57BL/6 (B6), cell-specific TLR4^−/−^, and global TLR4^−/−^ B6 mice were bred at our animal facility at the University of Pittsburgh and used at the age of 8–12 weeks. MyD88^−/−^ and Trif^−/−^ B6 mice were provided by Drs. Ruslan Medzhitov (Yale University) and Bruce Beutler (Scripps Research Institution), respectively. Transgenic B6 mice expressing Cre recombinase under control of the albumin (*Alb*), lysozyme (Lyz) or CD11c (*Cd11c*) promoters were obtained from The Jackson Laboratory (Bar Harbor, ME, USA). The contribution of TLR4 on myeloid cells and DC was determined by generating cell-specific TLR4^−/−^ B6 mice, including TLR4^loxP/loxP^ (Flox), Lyz-Cre × TLR4^loxP/loxP^, and CD11c-Cre × TLR4^loxP/loxP^ strains. Genotyping was performed using standard genomic PCR techniques.

### Mouse Model of Resuscitated Hemorrhagic Shock and Tissue Trauma (HS/T)

Mice, aged 8–12 weeks and weighing 20–30 g, were randomized into different control and experimental groups and anesthetized with pentobarbital sodium (80 mg/kg, i.p.), with repeated doses (10 mg/kg, i.p.) given when necessary during the experiment. A minimum of six animals were used for the experimental groups. This number was based on our extensive experience with the model and endpoints but also a power calculation that predicted (based on the variability in the model) that a 30% difference between groups should reach statistical significance. The HS/T model was performed as described previously by our group (29, 30). First, crushed bone fragments were prepared from humanely killed donor mice using the femurs and tibias of both hind limbs. The fragments were crushed into a bone mixture in 2 ml PBS using a sterile mortar and pestle. Additional mice were anesthetized, their limbs fixed against a warm operation plate to avoid low body temperature, and groin incisions were performed. The femoral arteries of both hind limbs were cannulated with a sterile PE-10 catheter, one for bleeding and the other was connected with a blood pressure monitor to record mean arterial pressure (MAP). Then both thighs were crushed for 30 s with a hemostat to induce soft tissue lesion, and the bone mixture from the donor mice was injected into the soft tissue injury area (0.15 ml/thigh) to induce a pseudofracture (PF) injury. Subsequently, blood was withdrawn through femoral artery cannula to induce HS (31); the MAP was maintained at 25 mmHg. After 2 h of HS/T, animals were resuscitated with Ringer’s lactate solution (LR) at 3 times the volume of shed blood. Mice were alive, kept warm with access to food and water after the HS/T model, and then euthanized at 3, 6, or 20 h following the initiation of resuscitation. The control group for the polytrauma mice was uninjured mice. This permitted us to compare baseline parameters (i.e., the preinjury state) to the accumulated impact of all of the acute insults commonly experienced by polytrauma patients, which include vessel cannulation, HS, severe tissue trauma, and anesthesia plus pain control. We have previously shown that the impact of these insults is indeed cumulative at the level of mRNA expression and the systemic inflammatory response (32, 33).

### Antibody Treatment

Anti-mouse TLR4 monoclonal antibody (mAb), 1A6, and its isotype-matched control mAb, W6/32, were provided by NovImmune SA (Geneva, Switzerland) and have been described previously (34). The B6 mice were pre-treated with 100 µg of 1A6 or its control mAB at 30 min prior to surgical intervention to initiate the HS/T model, and were euthanized for analysis at 3, 6, or 20 h after reperfusion.

### *In Vitro* Generation of DC

Dendritic cells were generated *in vitro* as previously described (35). Briefly, BM cells from the femurs and tibias of WT and TLR4^−/−^ B6 mice were depleted of erythrocytes with NH_4_Cl lysis solution. Erythroid cells, T and B lymphocytes, NK cells, and granulocytes were removed by incubation with the antibodies TER-119, CD3, B220, NK-1.1, GR1 followed by rabbit complement (Cederlane, Burlington, NC, USA). BM cells were cultured in RPMI-1640 with 10% FBS, glutamine, nonessential amino acids, sodium pyruvate, HEPES, 2-mercaptoethanol, pen strep, granulocyte-macrophage colony-stimulating factor (GM-CSF) (1,000 U/ml), and IL-4 (Peprotech, Rocky Hill, NJ, USA). Medium and cytokines were renewed every 2 days. Immature DCs were purified after 6 days of incubation by positive isolation with CD11c paramagnetic beads (Miltinyi Biotec; Auburn, CA, USA).

### Generation of Irradiation BM Chimeras

Male WT B6 mice (5–6 weeks old) were γ-irradiated with 550 rads twice, 6 h apart, and 2 h later were injected i.v. with 10^7^ BM cells from CD11c-diphtheria toxin (DT, 4 ng/g body weight) receptor (DTR), CD11c-TLR4^−/−^, or WT B6 mice (the latter to generate control B6 BM chimeras). Animals were kept in autoclaved cages, provided with sterile water with Sulfatrim during the first week, and used 8 weeks after BM infusion.

### Adoptive Transfer of DC

Adoptive transfer of BM-derived TLR4^+/+^ or TLR4^−/−^ DC into TLR4^−/−^ mice was performed as described previously (35): 10^7^ immature DC were adoptively transferred to either WT or TLR4^−/−^ B6 mice by tail vein injection with 300 µl of LR. The following recipient/donor combinations were produced: WT/WT, TLR4^−/−^/WT, and TLR4^−/−^/TLR4^−/−^. Tail vein injection of LR was used as a control for the adoptive transfer into TLR4^−/−^ mice.

### Flow Cytometry

For flow cytometric analyses, cell suspensions of DC generated *in vitro* were stained with a combination of the following fluorescently conjugated monoclonal antibodies: PE-CD3 (eBioscience), FITC-CD4, APC-CD8 (eBioscience), eFlour-450 CD11c (eBioscience), PE-NK1.1 (eBioscience), APC-Cy-7-Ly6G (eBioscience), FITC-MHC II (BD Biosciences). Samples were assayed on a BD LSR II flow cytometer (BD Biosciences) and data analyzed using FlowJo software (Tree Star, Ashland, OR, USA) as previously described (36).

### Lung and Liver Histology

Mice were euthanized after HS/T. The right upper lung lobe and the left lateral lobe of the liver were perfused with PBS and fixed in 2% paraformaldehyde (PFA). Tissues were then placed in 2% PFA for an additional 2 h and then switched to 30% sucrose in distilled water solution for 24 h. The tissue was then slowly frozen in liquid nitrogen cooled 2-methylbutane according to a standardized protocol for cryopreservation. Cryostat sections of the tissues (6 µm) were stained with hematoxylin and eosin (H&E) to evaluate histopathologic cumulative changes among treatment groups. Images of five randomly selected fields were acquired using an Olympus Provis light microscope (Malvern, NY, USA) with 200× magnification (37).

### Plasma Alanine Aminotransferase Assay

To assess hepatocellular injury, serum alanine aminotransferase (ALT) levels were measured using the Opera Clinical Chemistry System (Bayer Co.).

### Analysis of Inflammatory Mediators

Plasma IL-6 levels were detected with ELISA kits according to standard protocols. Inflammatory mediators were measured with a Luminex™ 100 IS apparatus (Luminex, Austin, TX, USA) using the BioSource 20-plex mouse cytokine bead set (BioSource-Invitrogen, San Diego, CA, USA) as per manufacturer’s specifications. The antibody bead kit included: GM-CSF, interferon-γ (IFN-γ), interleukin (IL)-1α, IL-1β, IL-2, IL-4, IL-5, IL-6, IL-10, IL-12p40, IL-12p70, IL-13, IL-17A, interferon-γ-inducible protein 10 (IP-10/CXCL10), keratinocyte-derived cytokine (KC/CXCL1), monocyte chemoattractant protein-1 (MCP-1/CCL2), monokine induced by interferon-γ (MIG/CXCL9), macrophage inflammatory protein-1α (MIP-1α/CCL3), tumor necrosis factor-α, and vascular endothelial growth factor. The final mediator concentrations are expressed in picograms per milliliter and presented as mean ± SEM.

### Statistical and Computational Analyses

The results are expressed as the mean ± SEM. All data were analyzed using SigmaPlot™ 12 software (Systat Software, Inc., San Jose, CA, USA). Statistical difference between groups was determined by either Student’s *t*-test, Mann–Whitney rank sum test, or two-way ANOVA as appropriate and indicated in the Figure Legends. *P* < 0.05 was considered statistically significant for all analyses.

Dynamic Bayesian Network (DBN) inference was used to model the evolution of the probabilistic dependencies within a system over time. This analysis was carried out using MATLAB™ (The Math Works, Inc., Natick, MA, USA), using an algorithm adapted from Grzegorczyk and Husmeier (38) and revised by our group (1, 39–44). In this analysis, inflammatory mediators were represented at multiple time points within the same network structure. Each node (inflammatory mediator) in the network was associated with a conditional probability distribution of a variable that is conditioned upon its parents (upstream nodes). This particular network structure was used to assess the dominant inflammatory mediators and the probable interaction among various mediators, including possible feedback loops (39, 41, 45).

## Results

### TLR4 Drives Systemic Inflammation and End-Organ Damage in a Mouse Model of HS/T

**Figure 1:**
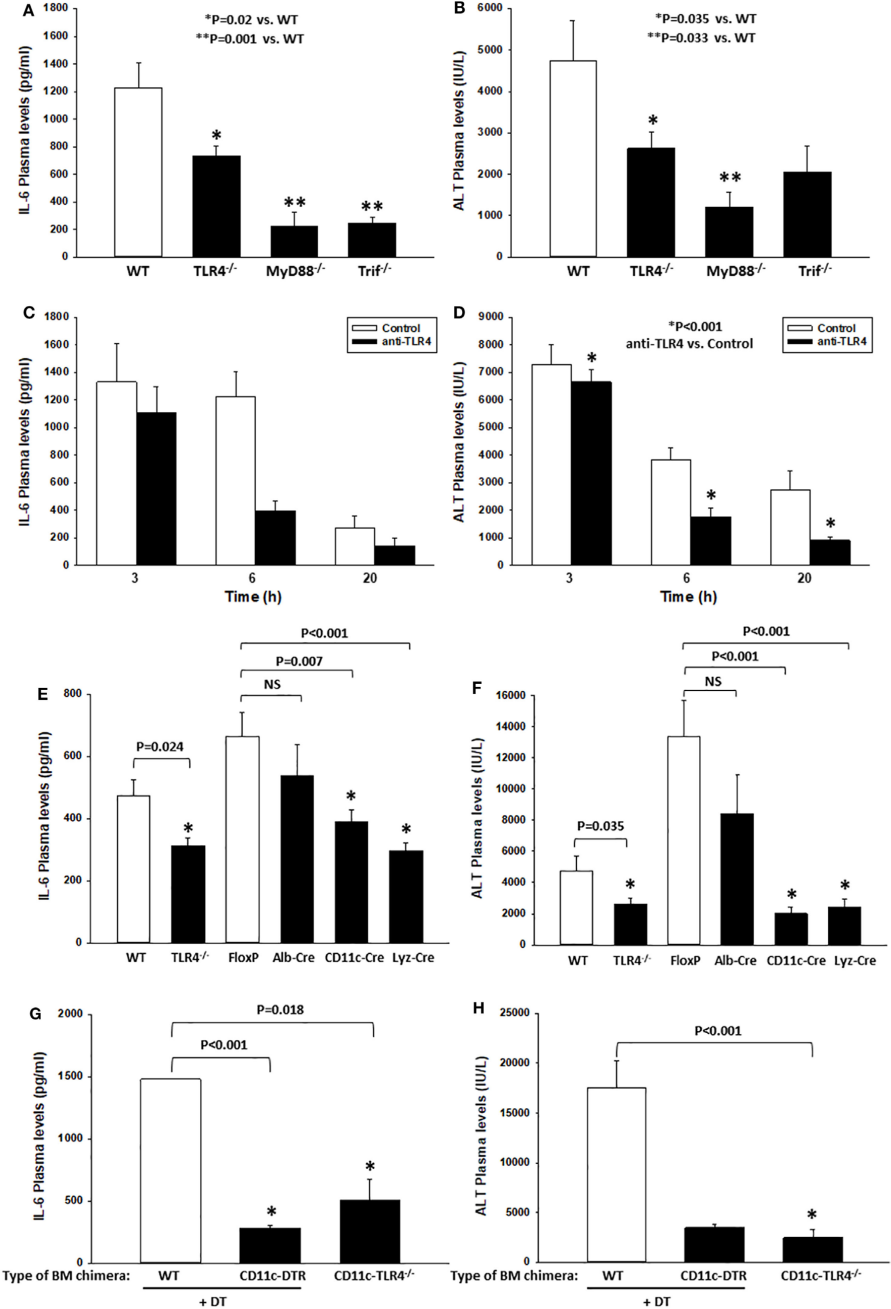
Toll-like receptor 4 (TLR4) drives systemic inflammation and end-organ damage in a mouse model of hemorrhagic shock (HS) and trauma (HS/T). Wild-type (WT), TLR4^−/−^, MyD88^−/−^, or Trif^−/−^ B6 mice were subjected to HS/T. IL-6 and ALT plasma levels were measured 6 h after the onset of HS/T as described in Section “Materials and Methods” **(A,B)**. In separate experiments, the selective anti-mouse TLR4 Ab 1A6 or its isotype control (W6/32 Ab) was administered intravenously 30 min prior to the initiation of the HS and the levels of IL-6 and ALT were measured at 3, 6, and 20 h after the onset of HS/T **(C,D)**. Panels **(A,C)** show IL-6 plasma levels, **(B,D)** show ALT plasma levels, **(E,F)** show IL-6 and ALT plasma levels after 6 h following the initiation of HS/T in the following cell-specific TLR4^−/−^ B6 mouse strains: Albumin-Cre × TLR4^loxP/loxP^ (Alb-Cre), CD11c-Cre × TLR4^loxP/loxP^ (CD11c-Cre), and Lyz-Cre × TLR4^loxP/loxP^ (Lyz-Cre). WT and TLR4^loxP/loxP^ (FloxP) mice were used as controls. Panels **(G,H)** show IL-6 and ALT plasma levels in lethally irradiated WT B6 mice reconstituted with bone marrow cells from CD11c-diphtheria toxin (DT) receptor (DTR) B6 mice or CD11c-TLR4^−/−^ B6 mice at 6 h after HS/T. WT B6 mice were used as controls [**(A,B,E,F)** *P < 0.05, analyzed by Mann–Whitney Rank Sum Test; **(C)** *P* = 0.050, **(D)** *P < 0.001, analyzed by two-way ANOVA; **(G,H)** *P < 0.001, analyzed by Student’s *t*-test (*n* = 6–15 animals/experimental condition)].

We (16) and others (46) have shown that TLR4 contributes to the inflammatory response in mouse models of HS or tissue trauma. Nearly all of these studies have relied upon genetic models in which TLR4 was either mutated or deleted. Here, we first confirmed that TLR4 is critical to the early systemic inflammatory response in a model involving both severe hemorrhage (HS for 2 h, MAP 25 mmHg) and severe extremity injury (bilateral PF, HS/T). In this experimental model, we have shown previously that plasma levels of IL-6, a common marker of the systemic inflammatory response syndrome, peak at 6 h (16). Likewise, liver damage assessed by circulating ALT levels as an indicator of end-organ damage also peaks at 6 h (16). In addition, we have shown that blocking IL-6 reduces the extent of liver damage, indicating that these two parameters are linked mechanistically in this model (30). Consistent with previous reports, we found significant increases in IL-6 (measured by ELISA) and ALT plasma levels at 6 h after the onset of HS/T in WT B6 mice (Figures 1A,B). In contrast, the plasma levels of these markers were much lower in TLR4^−/−^ B6 mice (Figures 1A,B) as well as in B6 mice deficient in MyD88, the proximal adapter protein required for TLR4-signaling. Similarly, in B6 mice deficient in Trif (another proximal adapter protein required for TLR4-signaling), IL-6 levels were significantly lower than those in WT B6 mice (Figure 1A), while plasma ALT levels trended lower than those of WT mice (Figure 1B). To establish that these findings were not unique to the genetic deletion of TLR4, we also tested if the selective anti-mouse TLR4 Ab 1A6 reduces systemic inflammation and end-organ damage in HS/T (Figures 1C,D). We first confirmed that the 1A6 Ab, but not the control isotype Ab (clone W6/32), decreased the activation of BM-derived CD11c^high^ DC induced by addition of either high mobility group box 1 (HMGB1) or LPS *in vitro* (data not shown). Pre-treatment with 1A6 Ab but not with its isotype control (W6/32 Ab) inhibited the increase in the plasma concentrations of IL-6 and ALT in the HS/T model (Figures 1C,D). Together, these results indicate that TLR4 and its downstream signaling pathways contribute to the early immune response and end-organ damage in this model of combined HS and tissue trauma.

### CD11c^+^ Cells Are Involved in the Early Systemic Inflammatory Response and Organ Damage following HS/T

We have previously used TLR4 BM chimeric mice to show that both BM-derived and non-BM-derived cells are involved in the TLR4-dependent systemic inflammatory response to either HS or extremity injury (9). Leukocytes of myeloid lineage, such as monocytes/macrophages and PMN, become activated and contribute to inflammation and organ injury in models of HS and tissue trauma (46, 47). However, we postulated that, in addition to myeloid cells, DCs contribute to the early response to sterile injury through TLR4-signaling. Therefore, we generated B6 mice that are TLR4-deficient in CD11c^+^ cells (DC-specific, DC-TLR4^−/−^), lysozyme (Lyz)^+^ cells (myeloid-specific, TLR4-myeloid^−/−^), or albumin^+^ cells (hepatocyte-specific, HC-TLR4^−/−^). Since hepatocytes are not expected to contribute significantly to the systemic inflammatory response to HS/T, we used HC-TLR4^−/−^ mice as controls. Other controls included TLR4^loxP/loxP^ mice. As shown in Figures 1E,F, TLR4 deletion from either CD11c^+^ or Lyz^+^ cells, but not albumin^+^ cells, resulted in significantly lower concentrations of IL-6 and ALT in plasma at 6 h following the initiation of HS/T than those detected in WT, TLR4^loxP/loxP^, or HC-TLR4^−/−^ mice. These reduced levels of IL-6 and ALT were similar to those found in the global TLR4^−/−^ mice. While the central role for myeloid leukocytes to TLR4-dependent responses was expected, the dominant role of CD11c^+^ cells indicated an unanticipated major function for DC in the initial phase of the systemic response to severe injury.

In addition to DC, several BM- and non-BM-derived cells express low levels of CD11c (48–52). Therefore, we tested in BM chimeras if BM-derived CD11c^high^ DCs, through TLR4, are critical to the injury-induced inflammatory response. To do that, we reconstituted lethally irradiated WT B6 mice with BM cells from the following donors: (i) CD11c-DTR B6 mice, to deplete selectively CD11c^high^ DC by DT injection; (ii) CD11c-TLR4^−/−^ (DC-TLR4^−/−^) B6 mice, to test the role of TLR4 expression exclusively on conventional DC; and (iii) WT B6 mice (used as controls). As shown in Figures 1G,H, control WT BM chimeras increased significantly the plasma levels of IL-6 and ALT at 6 h after HS/T as compared to CD11c-DTR BM chimeras, in which DC were depleted by DT injection, or to CD11c-TLR4^−/−^ BM chimeras, in which CD11c^high^ DC are deficient in TLR4. We conducted the experiments on BM chimeras because attempts to study CD11c-DTR mice directly were limited due to the high mortality of DT-treated CD11c-DTR mice following HS/T (not shown). Thus, BM-derived CD11c^+^ DC are major contributors to the systemic inflammatory response to HS/T.

**Figure 2:**
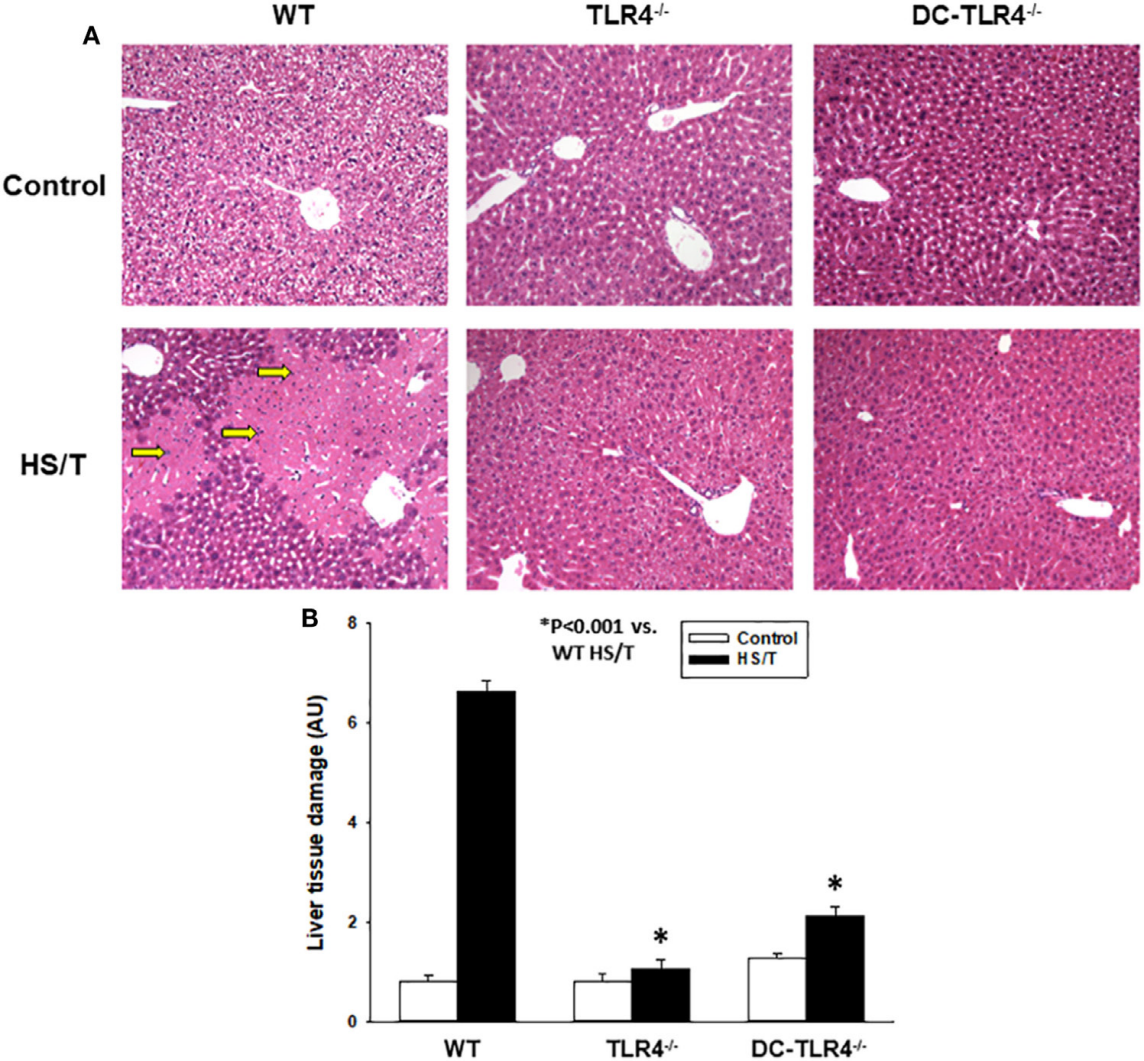
Histopathological analysis of liver in experimental bone marrow (BM) chimeras after HS/T. Liver sections from wild-type and experimental BM chimeras [TLR4^−/−^ and dendritic cell (DC)-TLR4^−/−^], left untreated (control) or 6 h after HS/T were stained with H&E **(A)** as described in Section “Materials and Methods.” Images are representative of 7–10 mice per treatment group. Arrows indicate areas of necrosis in liver. Panel **(B)** shows the quantification of organ damage (*P < 0.001, analyzed by Student’s *t*-test).

**Figure 3:**
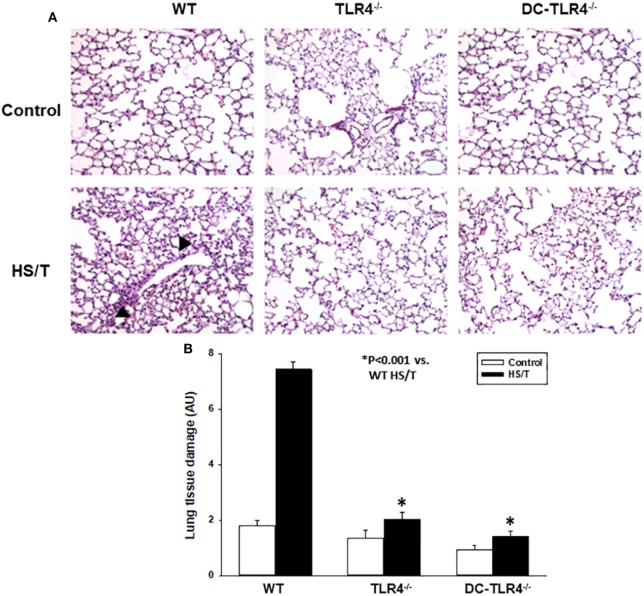
Histopathological analysis of lung in experimental bone marrow (BM) chimeras after HS/T. Lung sections from wild-type (WT) and experimental BM chimeras [TLR4^−/−^ and dendritic cell (DC)-TLR4^−/−^], left untreated (control) or 6 h after HS/T were stained with H&E **(A)** as described in Section “Materials and Methods.” Images are representative of 7–10 mice per treatment group. Arrow heads point to inflammatory cell infiltration in the lung. Panel **(B)** shows the quantification of organ damage (*P < 0.001, analyzed by Student’s *t*-test).

We further examined the extent of end-organ damage attributed to the TLR4 expression on CD11c^+^ DC by histopathological analysis of the livers and lungs from control and experimental BM chimeras, left untreated or 6 h after HS/T. As shown in Figures 2A,B, HS/T was associated to prominent central lobular necrosis in the liver, which is consistent with increased plasma levels of ALT and with our previous reports (9, 16, 53). Only negligible necrosis was detected in the livers of the DC-TLR4^−/−^ mice under similar conditions. Lung injury was likewise assessed by histologic scoring that evaluates septal thickening and leukocyte infiltrate. As expected, HS/T markedly increased the lung injury score in tissues analyzed 6 h after HS/T (Figures 3A,B). TLR4 deletion from CD11c^+^ cells nearly completely prevented the development of microscopic changes in the lungs following HS/T. Together, these data strongly support a role for CD11c^high^ DC in driving the TLR4-dependent inflammatory processes that cause lung and liver injury following HS/T.

### CD11c^high^ DC Contribute to Systemic Inflammation and Liver Damage after HS/T

**Figure 4:**
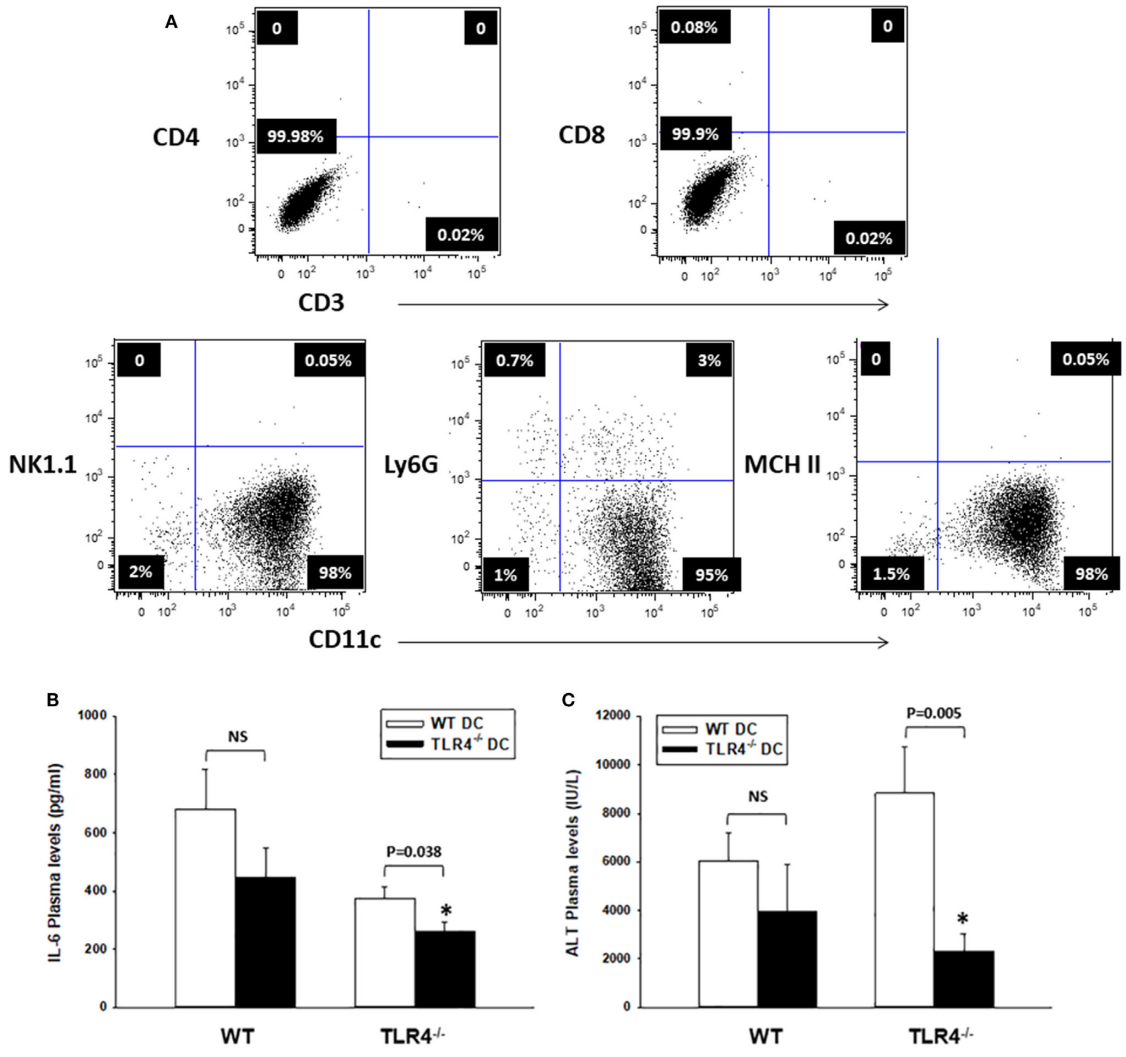
CD11c-dendritic cell (DC) contribute to systemic inflammation and liver damage after HS/T. **(A)** Flow cytometric analysis of TLR4^−/−^ DC generated from bone marrow precursors (day 6), purified by sorting with CD11c paramagnetic beads (Myltenyi), and adoptively transferred (i.v.) to TLR4^−/−^ [or wild-type (WT), control] B6 mice. The injected inoculum consisted mostly of immature DC (purity >95%). **(B,C)** TLR4^−/−^ B6 mice adoptively transferred (i.v.) with TLR4^−/−^ DC exhibited significantly lower concentrations of IL-6 and ALT in plasma measured 6 h after HS/T, as compared to TLR4^−/−^ mice injected with WT DCs. There were no significant differences in plasma concentrations of IL-6 and ALT following HS/T in WT B6 mice reconstituted (i.v.) with either WT or TLR4^−/−^ DCs **(B)** *P < 0.05, analyzed by Student’s *t*-test; **(C)** *P < 0.05, analyzed by Mann–Whitney Rank Sum Test (*n* = 8–12 independent experiments).

Bone marrow-derived leukocytes other than DC, such as macrophages, have been shown to express low levels of CD11c (50, 51). To confirm that CD11c^high^ DC promote the early inflammatory response post HS/T, we carried out experiments in host TLR4^−/−^ B6 mice reconstituted with WT and TLR4^−/−^ B6 DC transferred adoptively (i.v.). BM-derived DC were generated from BM precursors, depleted of erythroid cells, granulocytes, and T, B, and NK cells, and obtained from WT or TLR4^−/−^ B6 mice. CD11c^high^ DC were isolated by magnetic sorting (purity: 95% immature DC) (Figure 4A). Either WT or TLR4^−/−^ mice received 10^7^ DC (i.v.), 24 h prior to HS/T. In a separate experiment, green fluorescent protein (GFP)-expressing BM-derived DC were tracked in WT B6 mice 24 h following i.v. injection. GFP^+^ DC were detected in the lung, liver, and spleen after 24 h (Figure S1 in Supplementary Material), confirming the broad distribution and survival of the i.v. injected DC using this protocol.

We assessed systemic inflammation and liver damage, based on the plasma concentrations of IL-6 and ALT, at 6 h post HS/T. As shown in Figures 4B,C, adoptive transfer of TLR4^+/+^ DC, but not TLR4^−/−^ DC, led to a significant increase in the systemic inflammatory response and end-organ damage in the host TLR4^−/−^ B6 mice. No difference was detected in the plasma concentrations of IL-6 and ALT in control WT B6 mice reconstituted with TLR4^+/+^ or TLR4^−/−^ DC following HS/T. These data confirm that CD11c^high^ DC regulate the early systemic response to HS/T.

### Identification of the Early Drivers of Inflammation in the Mouse Model of HS/T

**Figure 5:**
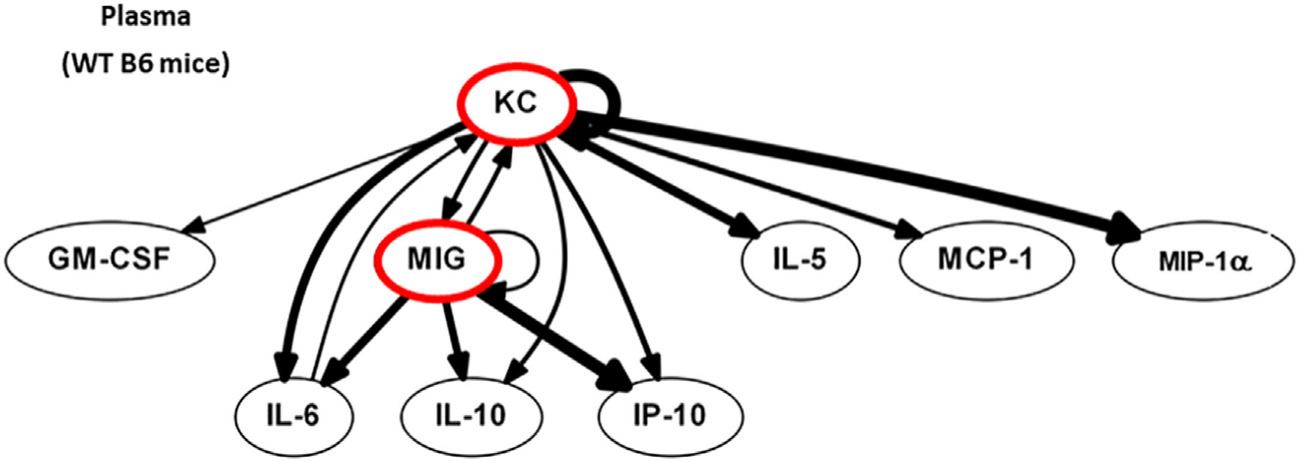
MIG and KC are early drivers of inflammation in a mouse model of HS/T. Dynamic changes in 20 cytokines and chemokines were measured in wild-type (WT) B6 mice subjected to HS/T at 0, 2 (end of shock), 3, 6, and 24 h as described in Section “Materials and Methods.” Using the time courses of circulating inflammatory mediators, Dynamic Bayesian Network (DBN) inference was used to determine possible network structures that exhibit self-feedback as central nodes. DBN inference suggested a primary network driven with core motifs consisting of MIG and KC, each of which drives its own expression and were inferred to affect the downstream production of IP-10, MCP-1, and IL-10 (*n* = 6 independent experiments).

We next postulated that DC, by sensing local tissue stress or damage, would regulate the earliest phases of immune activation after injury. Computational strategies can be used to identify the most proximal and dominant drivers of inflammation if dynamic changes are measured (54). Dynamic changes in 20 circulating protein mediators (see Figure S2 in Supplementary Material) were measured in WT B6 mice at 0, 2 (end of shock), 3, 6, and 24 h. First, we identified feedback structures and possible regulatory architectures in dynamic networks of inflammation in WT B6 mice subjected to HS/T as described in the Section “Materials and Methods.” We utilized DBN inference to determine if such network structures could be discerned from the time courses of circulating inflammatory mediators. We focused primarily on mediators that exhibit self-feedback as central nodes, as in our recent studies in trauma, sepsis, and liver failure (1, 39, 41, 44, 45). DBN inference suggested a primary network driven with core motifs consisting of the CXC chemokines MIG and KC, each of which drives its own expression. Those central nodes were inferred to affect the downstream production of different mediators including IP-10, MCP-1, and IL-10 (Figure 5).

### TLR4 on CD11^high^ DC and Myeloid Cells Is Required for KC, MIG, and IP-10 Increases following HS/T

**Figure 6:**
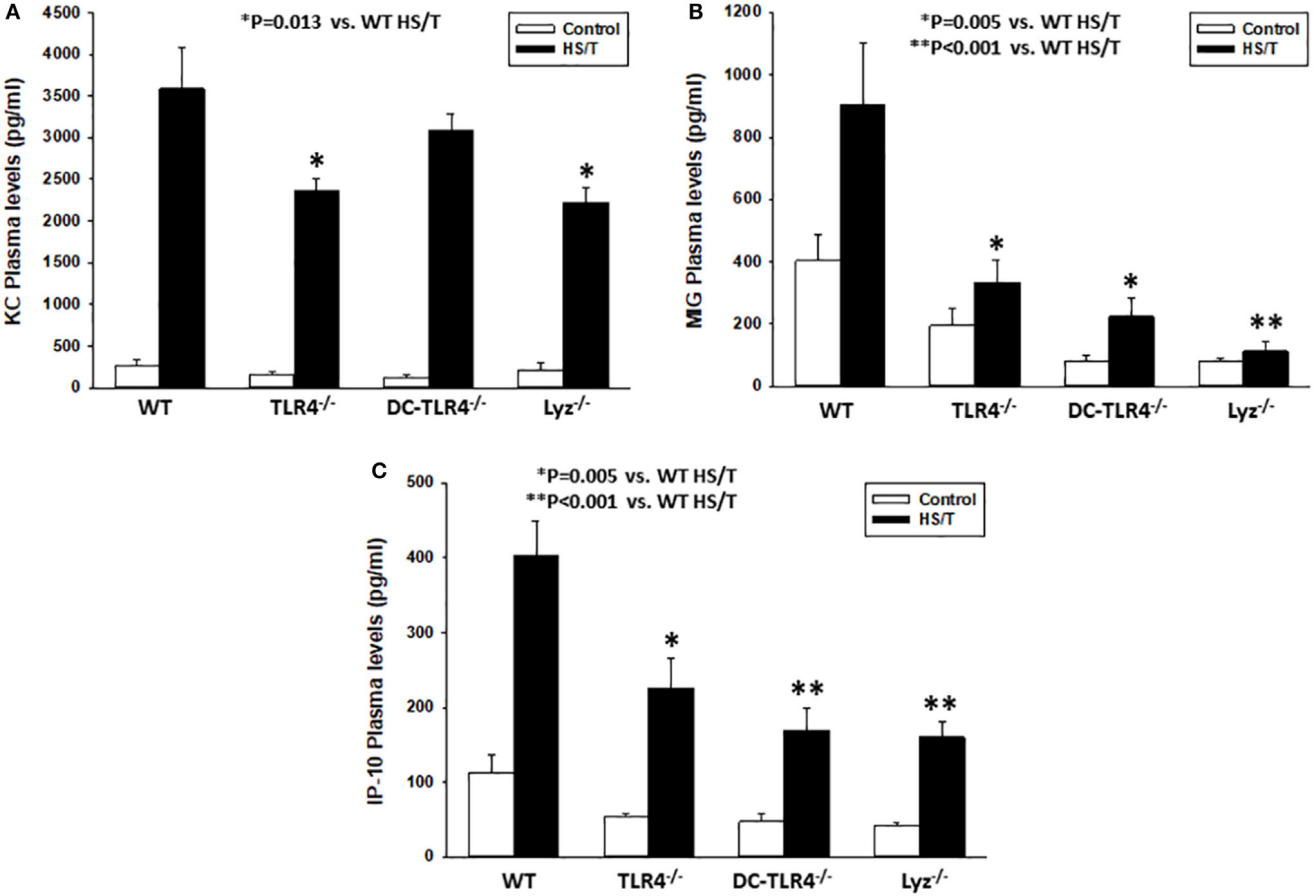
Toll-like receptor 4 (TLR4) on CD11^high^ dendritic cell (DC) and myeloid cells is required for KC, MIG, and IP-10 increases following HS/T. Mice where TLR4 was selectively deleted from CD11c^+^ or Lyz^+^ cells were subjected to HS/T and plasma levels of KC **(A)**, MIG **(B)**, and IP-10 **(C)** were measured by Luminex as described in Section “Materials and Methods.” All three chemokines levels were significantly lower in mice subjected to HS/T where TLR4 was selectively deleted from CD11c^+^ or Lyz^+^ cells. MIG and IL-10, but not KC, were lower in DC-TLR4^−/−^ mice subjected HS/T [*P < 0.05, analyzed by Student’s *t*-test (*n* = 7–13 independent experiments)]. Comparison of the basal levels (control) showed, with one exception, no statistical significant differences between the different strains as compared to wild-type (WT) [**(A)** WT vs. TLR4^−/−^ (*P* = 0.228), DC-TLR4^−/−^ (*P* = 0.207), Lyz^−/−^ (*P* = 0.760); **(B)** WT vs. TLR4^−/−^ (*P* = 0.067), DC-TLR4^−/−^ (*P* = 0.013), Lyz^−/−^ (*P* = 0.059); **(C)** WT vs. TLR4^−/−^ (*P* = 0.057), DC-TLR4^−/−^ (*P* = 0.079), Lyz^−/−^ (*P* = 0.145)].

Our DBN analysis identified the prominent role of CXC chemokines as proximal drivers of the systemic inflammatory response in our mouse model of HS/T. To determine if CD11c^high^ DC and/or leukocytes of myeloid lineage contribute to the elevations in CXC chemokines, we measured the concentrations of KC (Figure 6A), MIG (Figure 6B), and IP-10 (Figure 6C) in plasma in our experimental groups. Comparison of the basal levels (control) of these chemokines showed, with one exception, no statistical significant differences between the different strains as compared to WT. In contrast, all three chemokines levels were significantly lower in mice subjected to HS/T when TLR4 was selectively deleted from CD11c^+^ or Lyz^+^ cells. MIG and IP-10, but not KC, were lower in DC-TLR4^−/−^ mice subjected to HS/T (Figure 6). Thus, TLR4 on DC and myeloid leukocytes selectively contributes to the increases in the protein mediators identified computationally as the most proximal drivers of inflammation in our mouse models of HS/T.

## Discussion

Toll-like receptor 4 is an important driver of the innate and adaptive immune responses in experimental models that simulate polytrauma in humans (9, 16, 21). This study was undertaken to understand the contribution of TLR4 on leukocytes of myeloid lineage and conventional (CD11c^high^) DC in the initial systemic inflammatory response induced by trauma with HS. As expected, we found that TLR4 on myeloid leukocytes contributes significantly to release of cytokines and chemokines into the systemic circulation and early organ damage after HS/T. Perhaps less expected was the dominant role of TLR4 on conventional DC in this response. We also show that TLR4 on both DC and myeloid cells is required for the elevations in the concentrations of CXC chemokines in plasma. These chemokines were found to be among the most proximal mediators in dynamic inflammatory networks identified by measuring circulating chemokine and cytokine levels in this model. Therefore, conventional DC and leukocytes of myeloid lineage appear to be critical to the initial sensing of tissue damage and stress through TLR4 during HS/T.

The importance of DC subsets in regulating adaptive immunity is well established. However, the contribution of DC in sensing tissue damage or stress during severe systemic injury and then regulating systemic inflammatory responses is less clear. Data have begun to accumulate from studies in human trauma patients suggesting that DC are activated early and exhibit an increased inflammatory gene expression (55). This includes the upregulation of mRNA for CXC chemokine family members, including CXCL5 and CXCL4 (27, 28). These changes, as well as a decline in total monocyte-derived DC numbers, were detected as early as 1 day after severe injury in humans. The decrease in monocyte-derived DC numbers was thought to be due to increased apoptosis (55).

Studies in murine models of HS with or without tissue trauma have been centered on the analysis of antigen-presenting properties of splenic DC (26) or immunoregulatory roles of CD103^+^ DC in the gut (56). Splenic DC numbers, maturation, MHC class II expression, and antigen presentation are all suppressed after HS plus tissue trauma (26). The numbers of intestinal CD103^+^ DC are also suppressed after HS in rats (56).

In contrast to these studies, we identified a role for TLR4 on conventional CD11c^high^ DC in promoting CXC chemokine and cytokine production in a model of HS/T. Therefore, while the number and function of some populations of DC are depressed following systemic injury in mice, TLR4 stimulation on conventional immature DC contributes to promoting systemic inflammation in the initial stages of the sterile inflammatory response induced by HS/T. This finding is consistent with our previous observation that the expansion of DC numbers in the liver promotes TLR4-dependent hepatic damage in the model of liver ischemia and reperfusion (57).

We used dynamic changes in circulating levels of 20 inflammatory mediators to identify the proximal drivers of inflammatory networks in this mouse model of HS/T using computational modeling. DBN inference revealed that CXC chemokines, including KC and MIG, represent central and early nodes in the networks that appear to be upstream of IL-6, IL-10, and IP-10 in the circulation. KC and MIG production by liver macrophages has been demonstrated in mouse injury models, and these mediators have been shown to contribute to the pro-inflammatory response and liver damage (2, 58, 59). He, we demonstrate that TLR4 on conventional DC is required for increase in circulating levels of MIG and IP-10, while TLR4 on myeloid cells contributes to elevations in KC, MIG, and IP-10. It has been known for some time that DC release significant quantities of CXC chemokines in response to TLR agonists (60, 61). This, in addition to work showing that human monocyte-derived DC upregulates CXC chemokine mRNA levels early after injury, suggests that DC are an important source of these early drivers of systemic inflammation after HS/T in humans. However, the differential dependence of TLR4 on conventional DC and myeloid cells for KC elevations suggests that the early production of this chemokine is more dependent on myeloid cells and that the interaction between DC and myeloid cells is an important part of the early response to systemic injury.

It is now well established that a subset of TLR are important regulators of the sterile inflammatory response. In addition to TLR4, these include TLR2, TLR3, and TLR9 (4–8) while the cell-selective and specific function of each receptor has not been shown, all appear to participate in the increase in the inflammatory response to sterile injury in various models. TLR3 and TLR9 recognize only nucleic acids (TLR3 binds to dsRNA and TLR9 to hypomethylated DNA). These observations indicate that the recognition of molecules spans a range of host ligands from nucleic acids to proteins. For TLR4, TLR2, and TLR9, receptors on both BM-derived and non-BM-derived cells are involved (9, 10). More work is needed to define the role of specific pattern recognition receptors on the many cell types that express TLRs in the systemic inflammatory response to injury.

Toll-like receptor 4 recognizes multiple self-derived molecules that contribute to inflammation in murine HS/T models. These include HMGB1, hyaluronan, and heat-shock proteins (5, 8, 11, 14, 15). The specific ligands that trigger signaling of TLR4 on DC were not identified in this study, but could involve multiple ligands released by dying or stressed cells. It is likely that macrophages and DC act in parallel in promoting the earliest stages of inflammation after trauma. However, our findings do not distinguish the timing or selective roles of these cell types. That deletion of TLR4 on either cell type was sufficient to suppress systemic inflammation after injury indicates that a coordinated response requires both cell types. Our observation that transferring only 10^7^ TLR4^+^ DC into global TLR4^−/−^ mice was sufficient to partially reconstitute the systemic response to injury suggests that relatively few CD11c^high^ DC are required to regulate the innate immune response to a severe systemic stress due to trauma.

Toll-like receptor 4 remains a potential therapeutic target to prevent an exaggerated systemic inflammatory response after injury. We have shown that a TLR4 antagonist given early after injury prevents liver injury after HS (53). In this study, we show that administration of a highly selective Ab against TLR4 also blocks systemic inflammation and liver damage in a model involving not only severe HS but also severe peripheral tissue trauma. The current work, in combination with previous studies on the role of TLR4 in regulating the adverse immune response to injury (19, 21–24, 26), suggest that TLR4 blockade should be considered for clinical trials in trauma patients prone to organ dysfunction.

## Ethics Statement

This study was carried out in accordance with the recommendations of the National Institutes of Health Guidelines for the Use of Laboratory Animals. The protocol was approved by the Animal Care and Use Committee of the University of Pittsburgh.

## Author Contributions

KZ and SK participated in experimental design, animal and cell culture experiments, and writing. RZ participated in computational and statistical analysis, data interpretation, and writing. AM participated in experimental design, data interpretation, and writing. SD participated in data collection and analysis. PL participated in histological analyses. GE and SS-P provided reagents. LS provided reagents and participated in data interpretation. MS participated in experimental design and data interpretation. YV participated in data interpretation and writing. TB participated in study design, data interpretation, and writing.

## Conflict of Interest Statement

The authors declare that the research was conducted in the absence of any commercial or financial relationships that could be construed as a potential conflict of interest.

## Supplementary Material

Figure S1 Selective localization of dendritic cell (DC) detected by immunostaining of lung, spleen, liver, kidney, and mesenteric and peripheral lymph nodes (LN) sections. Green fluorescent protein (GFP)-expressing bone marrow-derived DC were tracked in lung, spleen, liver, kidney, and mesenteric and peripheral LN of wild-type B6 mice 24 h following i.v. injection. GFP^+^ DC were detected only in the lung, liver, and spleen.

Figure S2 Measurement of cytokines/chemokines by Luminex. Dynamic changes in 20 cytokines and chemokines were measured in plasma from wild-type (WT) B6 mice subjected to HS/T at 0, 2 (end of shock), 3, 6, and 24 h as described in Section “Materials and Methods.” The final mediator concentrations are expressed in picograms per milliliter.
