# Supplementary figures and images for: Toll-Like Receptor 4 on both Myeloid Cells and Dendritic Cells Is Required for Systemic Inflammation and Organ Damage after Hemorrhagic Shock with Tissue Trauma in Mice
Source: Front Immunol. 2017 Nov 28;8:1672. doi: 10.3389/fimmu.2017.01672 (PMC5712321; doi:10.3389/fimmu.2017.01672)

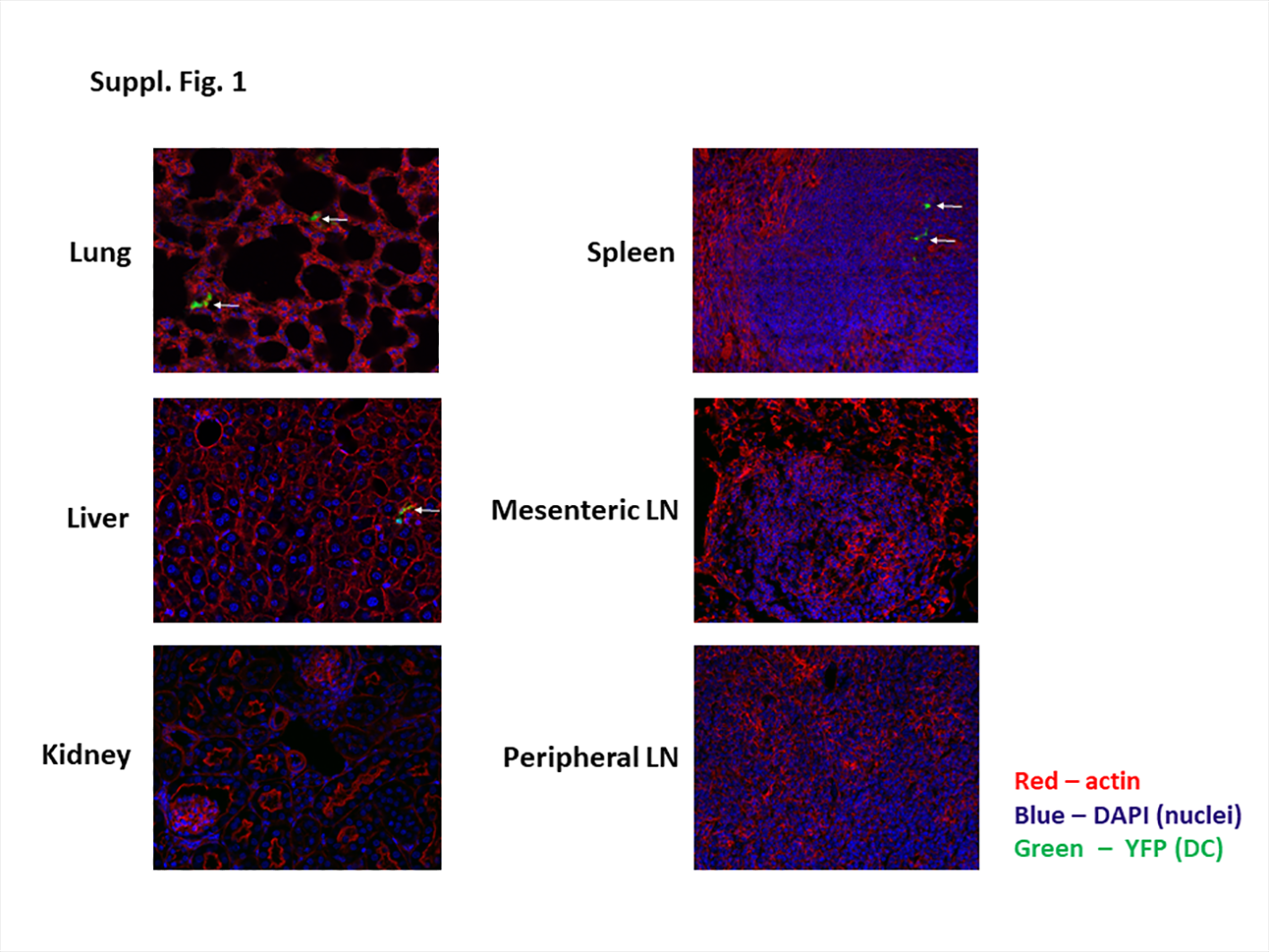

Supplement: Figure S1 — Selective localization of dendritic cell (DC) detected by immunostaining of lung, spleen, liver, kidney, and mesenteric and peripheral lymph nodes (LN) sections. Green fluorescent protein (GFP)-expressing bone marrow-derived DC were tracked in lung, spleen, liver, kidney, and mesenteric and peripheral LN of wild-type B6 mice 24 h following i.v. injection. GFP+ DC were detected only in the lung, liver, and spleen. [file Image_1.tif]

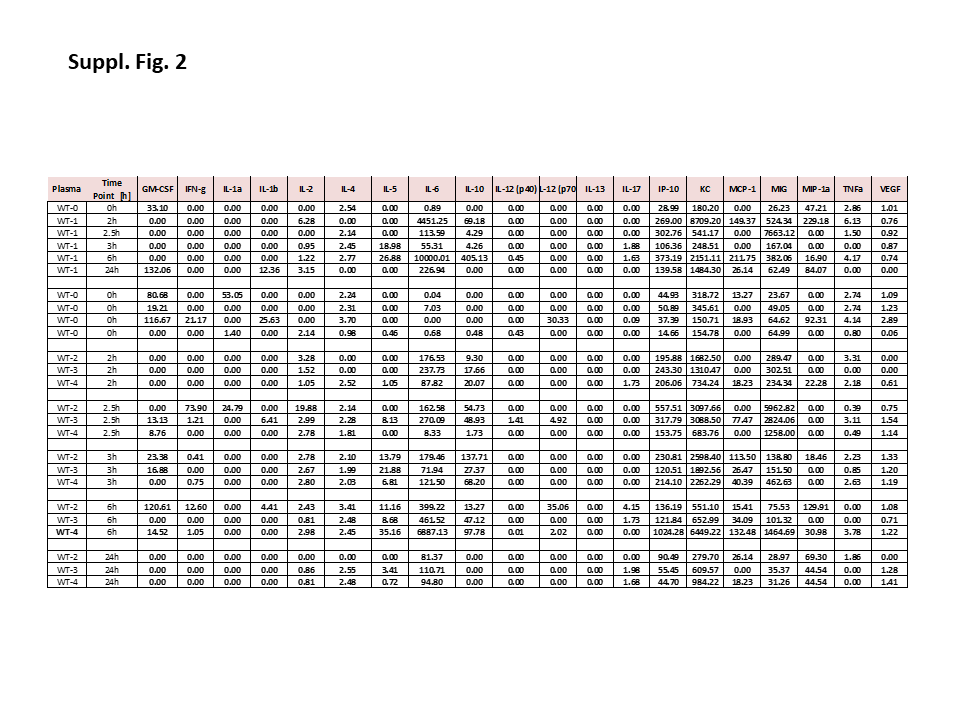

Supplement: Figure S2 — Measurement of cytokines/chemokines by Luminex. Dynamic changes in 20 cytokines and chemokines were measured in plasma from wild-type (WT) B6 mice subjected to HS/T at 0, 2 (end of shock), 3, 6, and 24 h as described in Section “Materials and Methods.” The final mediator concentrations are expressed in picograms per milliliter. [file Image_2.tif]
